# Supplementary material for: Cryo-EM study of an archaeal 30S initiation complex gives insights into evolution of translation initiation
Source: Commun Biol. 2020 Feb 6;3:58. doi: 10.1038/s42003-020-0780-0 (PMC7005279; doi:10.1038/s42003-020-0780-0)
Supplement: Supplementary file 1 — Supplementary Information [file 42003_2020_780_MOESM1_ESM.pdf]

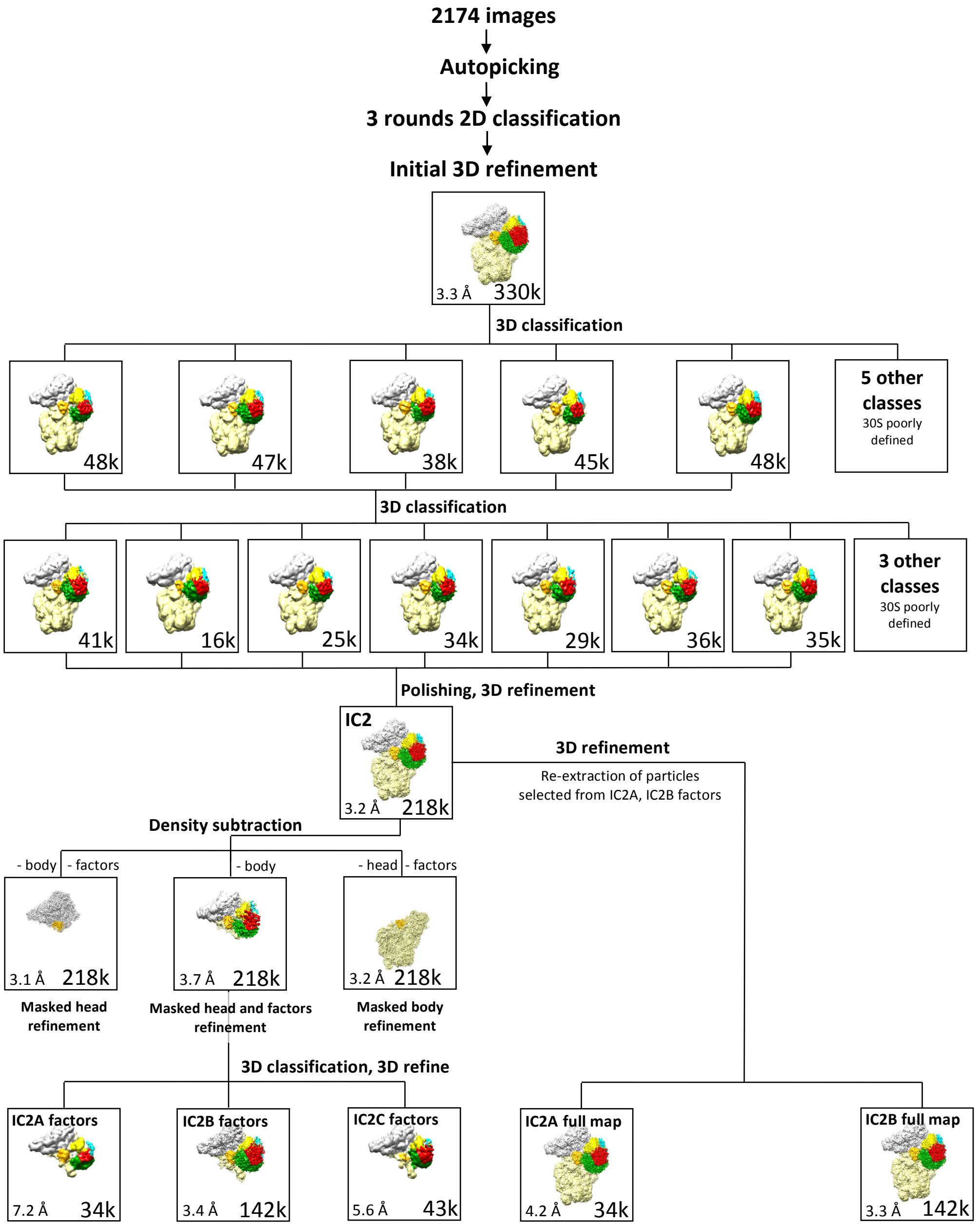

Supplementary Fig. 1: Data processing flowchart of IC2 cryo-EM dataset. Several steps of 3D classification, polishing and density subtraction using RELION allowed us to identify two conformations: IC2A (4.2 Å resolution) and IC2B (3.3 Å resolution). The head and body parts of the 30S were refined at high resolution (3.1 and 3.2 Å resolution respectively). The initiation factors bound to the 30S subunit (head in grey and body part in dark yellow) are colored as follows: aIF2γ green, aIF2α cyan, aIF2β red, aIF1A orange and tRNA bright yellow.

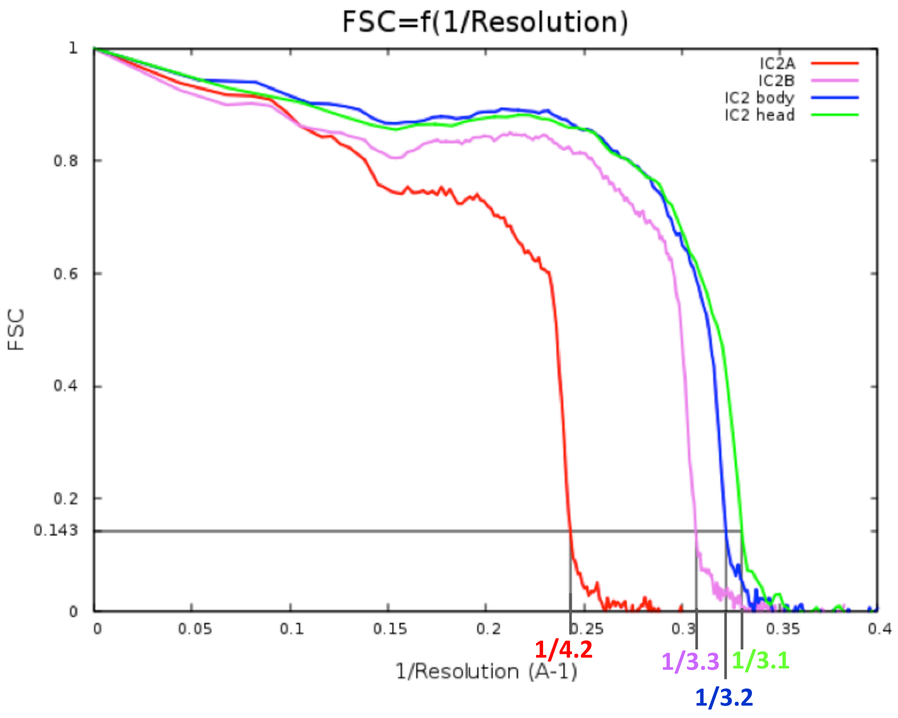

Supplementary Fig. 2: Fourier shell correlation curves of calculated maps. IC2A map is in red, IC2B in purple, IC2 body in blue and IC2 head in green. The overall resolutions of the different reconstructions , estimated using the FSC = 0.143 criterion[1], are 4.2 Å, 3.3 Å, 3.2 Å and 3.1 Å for IC2A, IC2B, IC2 body and IC2 head respectively.

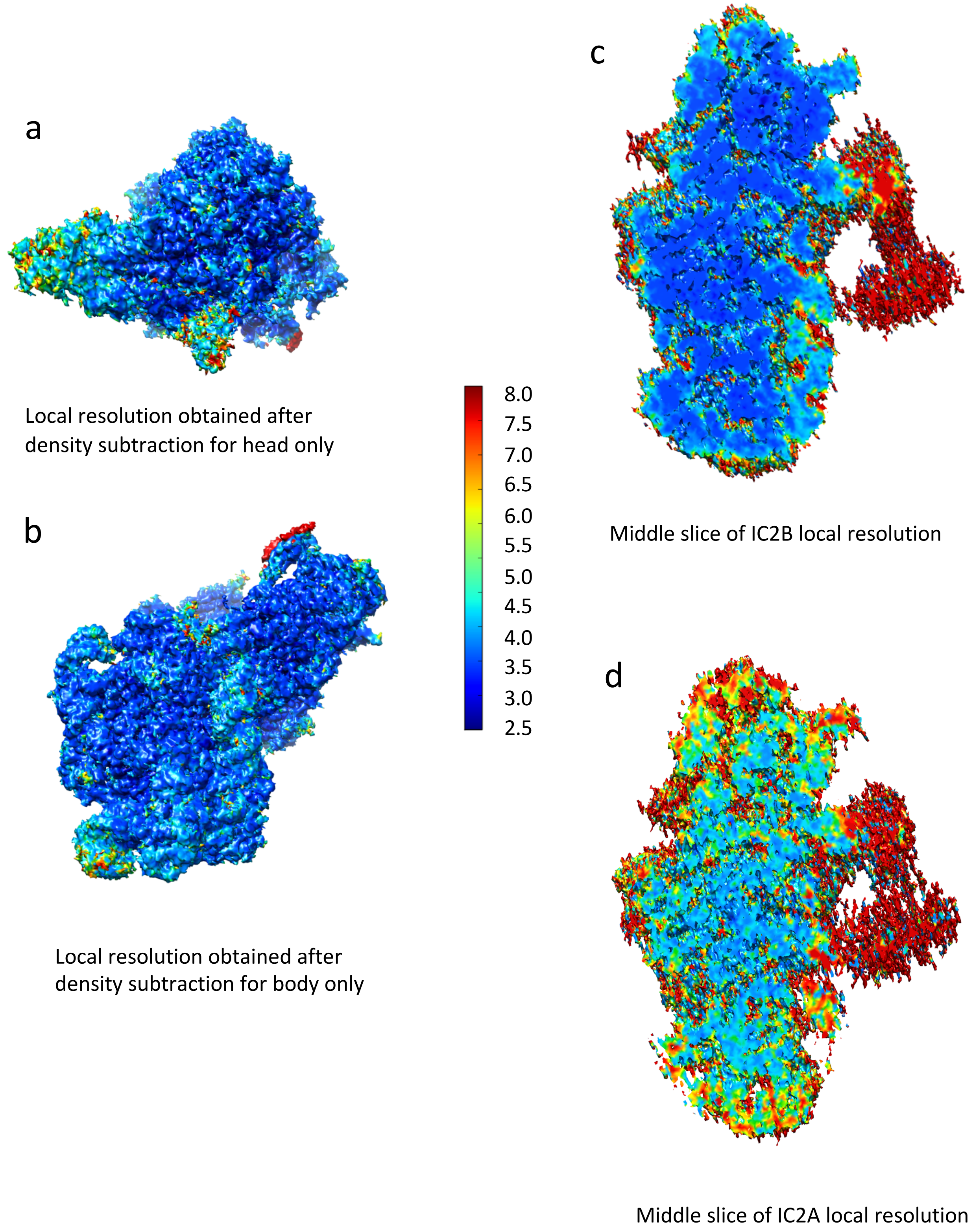

Supplementary Fig. 3: Local resolution maps. Local resolution is calculated with Resmap[2] of IC2 head (a), IC2 body (b), IC2B (c), IC2A (d) ranging between 2.5 Å in blue to 8 Å in red. The overall local resolution maps were shown for IC2 head and IC2 body whereas a central cut of the map was shown for IC2B and IC2A to distinguish the structured core of the 30S subunit compared to the more flexible part of the bound factors.

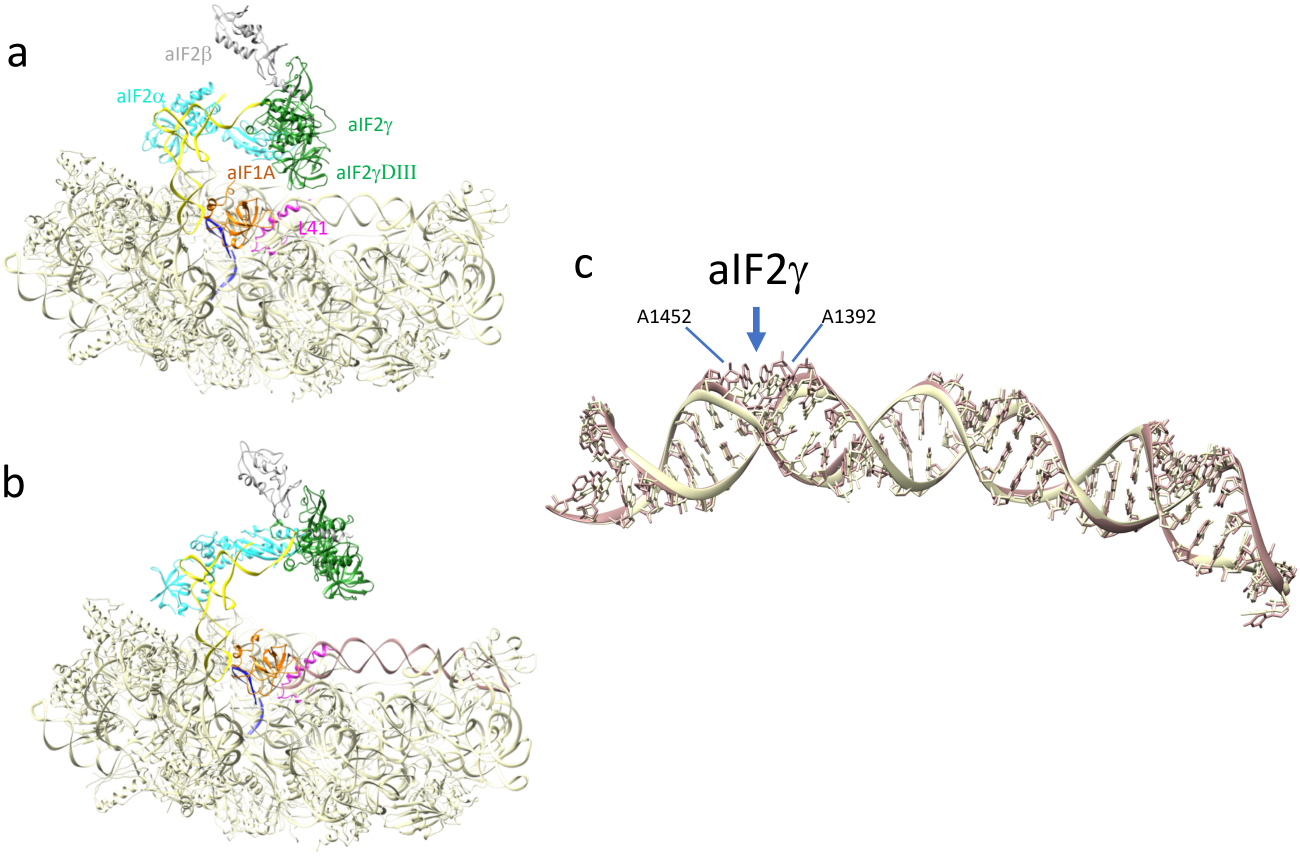

Supplementary Fig. 4: adjustment of h44 conformation upon aIF2γ release. **a**, IC2A model. **b**, IC2B model, **c**, h44-IC2A (pale yellow) superimposed on h44-IC2B (rosy brown). The aIF2γDIII binding site is indicated.

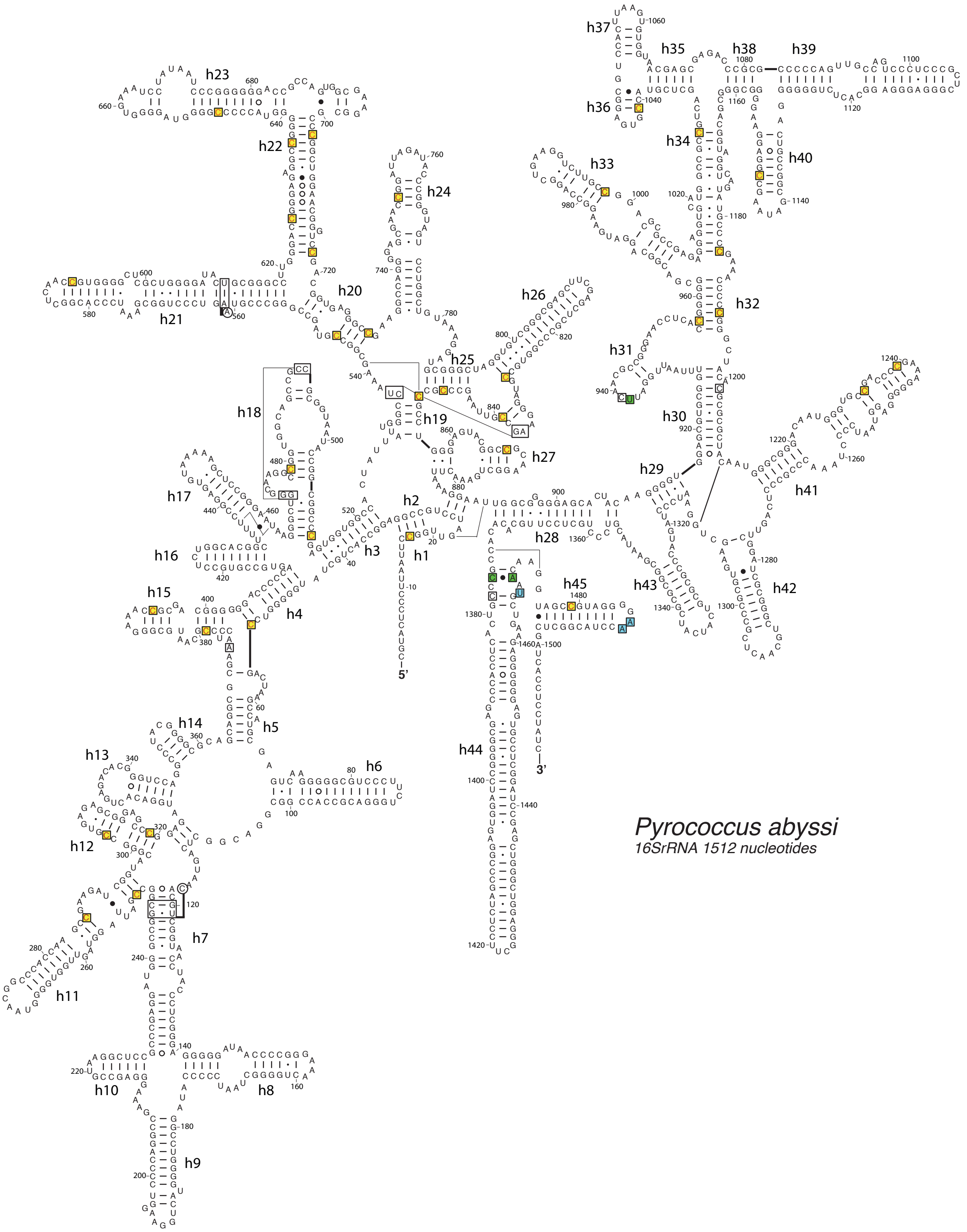

Supplementary Fig. 5: Secondary structure diagram of the *P. abyssi* 16S rRNA. The *P. abyssi* 16S rRNA diagram was updated from the diagram published on http://www.rna.icmb.utexas.edu/[3]. Modified nucleotides as indicated in Tables 3 and 4 are individually boxed. N^4 acetylcytidines are in red with yellow background.

a

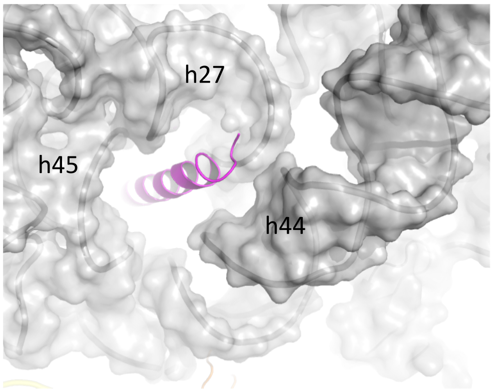

b

AAGGAGGTGACGCTCATGAAGAGGAGGCCAAGGAAGTGGAAGAAGAAGGGTAGAATGAGGTGGAAGTGGATTAAGAAGAGGATAAGGAGGCTCAAGAGACAGAGAAAGAAGGAGAGAGGACTGCTCTAA

M K R R P R K W K K K G R M R W K W I K K R I R R L K R Q R K K E R G L L

c

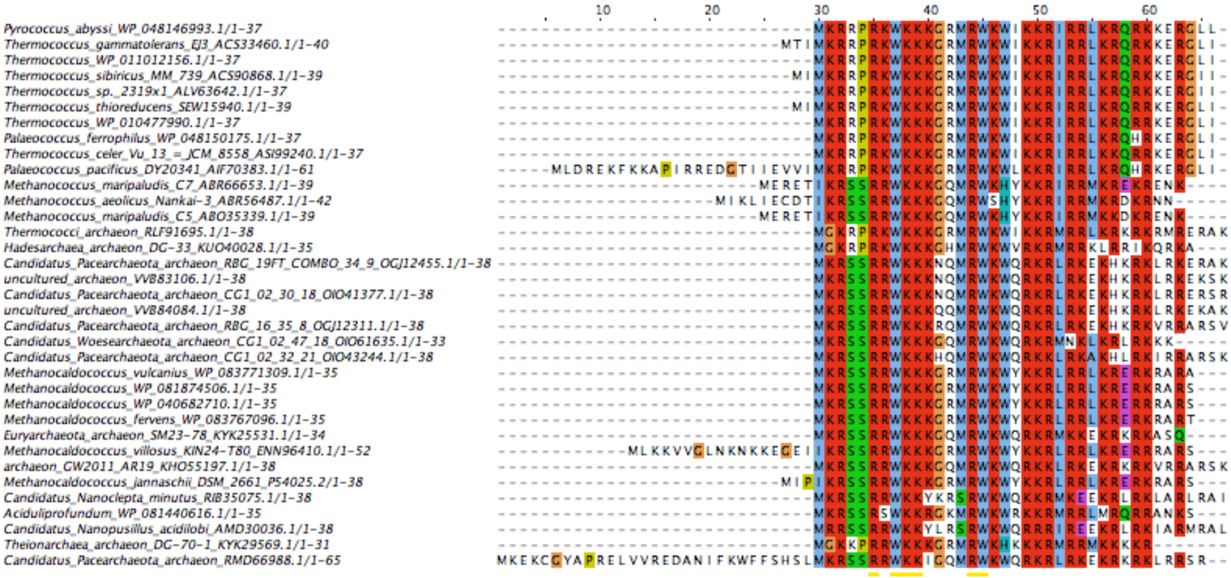

Supplementary Fig. 6: Identification of aL41 from *Pyrococcus abyssi*. **a**, aL41 binding site on Pab-30S. aL41 is in magenta cartoon and the remaining part of the ribosome is represented as a transparent molecular surface with gray cartoon. **b,** The nucleotidic sequence of ORF PAB_RS07520 (NCBI reference) identified as hypothetical protein in the *Pyrococcus abyssi GE5* genome is shown. SD sequence, ATG in frame codons and stop codon are in red. **c**, Sequence alignment of putative aL41 proteins as identified through BLAST search against archaeal genomes using the *P. abyssi* aL41 sequence. The clustal color code is used.

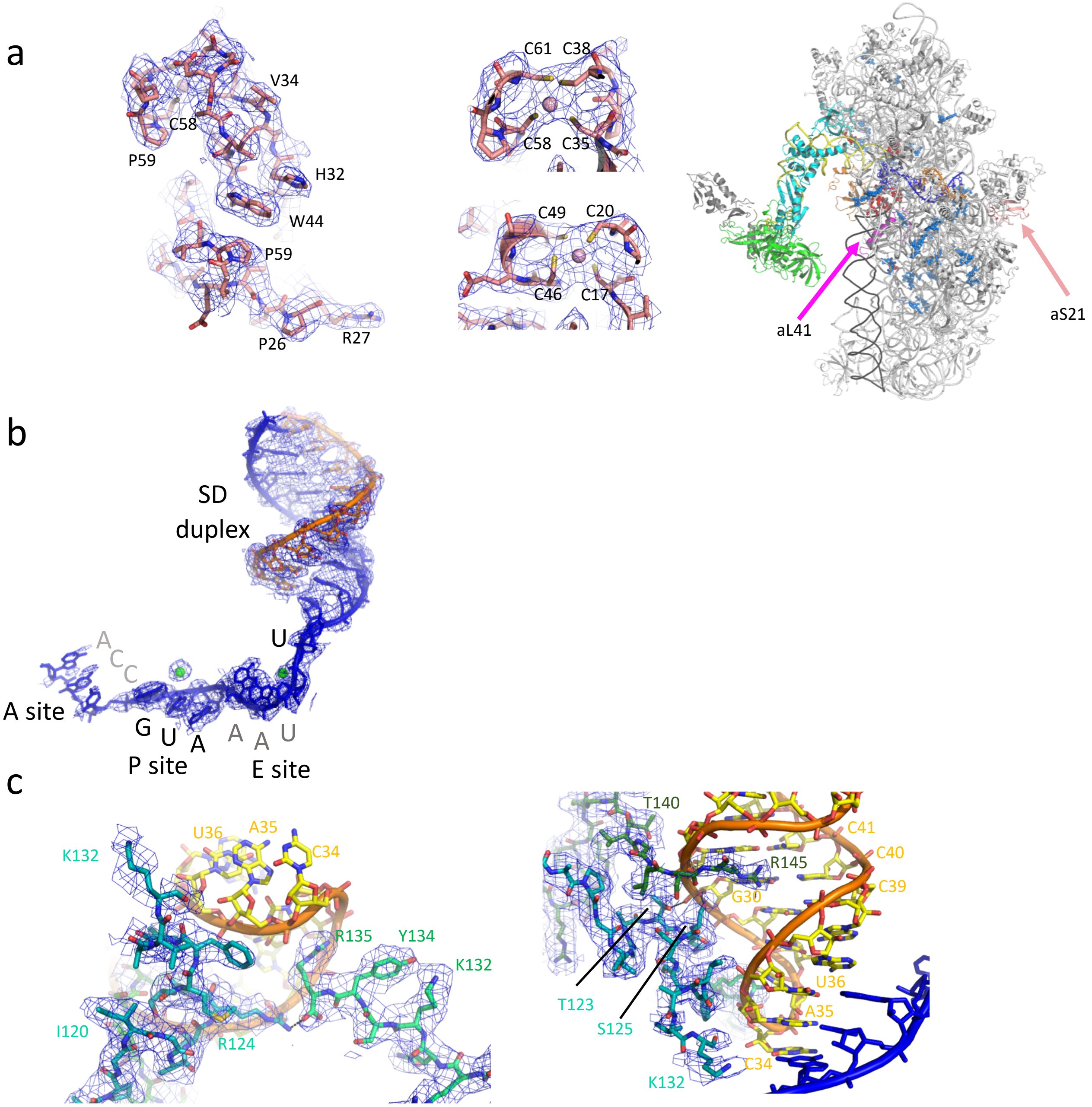

Supplementary Fig. 7: **Cryo-EM maps in regions discussed in the text. a**, Cryo-EM map of parts of aS21. Locations of aS21 (pink) and aL41 (magenta) on the 30S are indicated by arrows in the right view. **b**, Cryo-EM map around mRNA and the SD duplex in the same orientation as Fig. 3a. **c**, Cryo-EM map around uS9, uS13 and uS19. left, the C-terminal tails of uS9 (light green) and uS19 (dark cyan) are shown. right; the C-terminal tails of uS19 (dark cyan) and uS13 (dark green) are shown. The initiator tRNA is in orange with yellow sticks. mRNA is in blue. The electron density map is represented in blue mesh at 5 σ (views a and b) and 3 σ (views c) using the carve command in Pymol. Some residues are labeled.

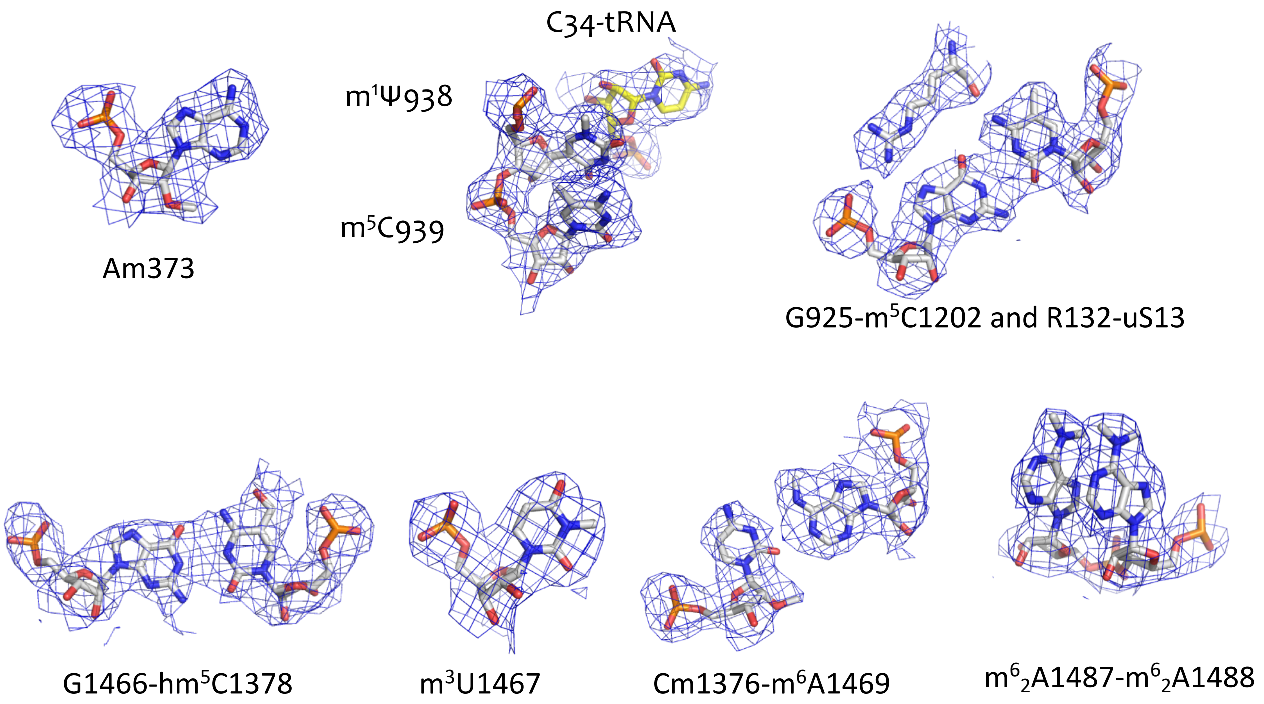

Supplementary Fig. 8: Cryo-EM map for modified nucleotides as described in Table 3. The electron density map is represented in blue mesh at 5σ using the carve command in Pymol.

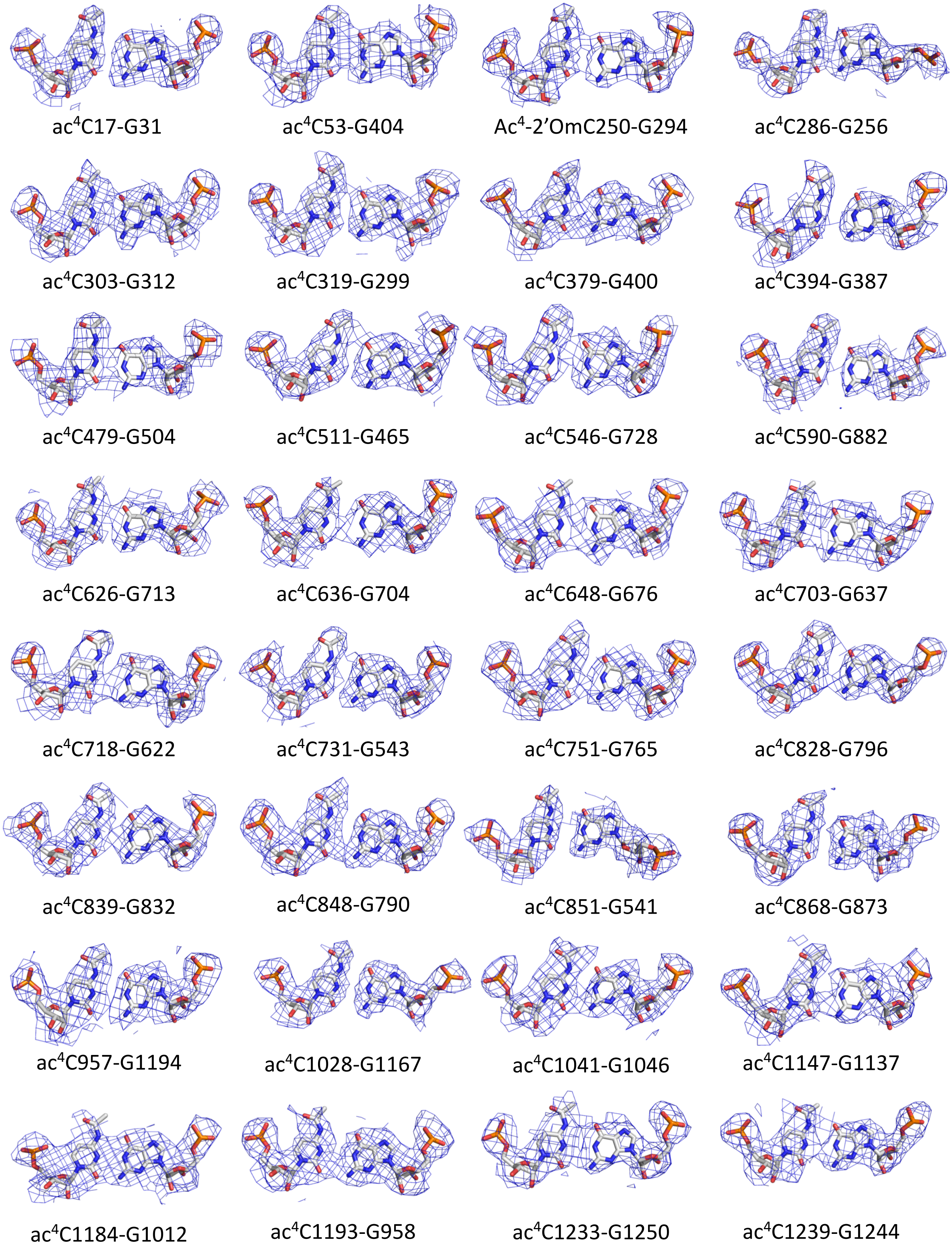

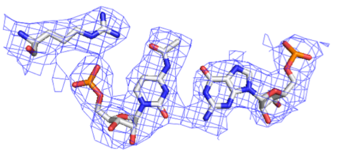

Supplementary Fig. 9: Cryo-EM map for N^4-acetylcytidines as described in Supplementary Table 2. The electron density map is represented in blue mesh at 5σ using the carve command in Pymol.

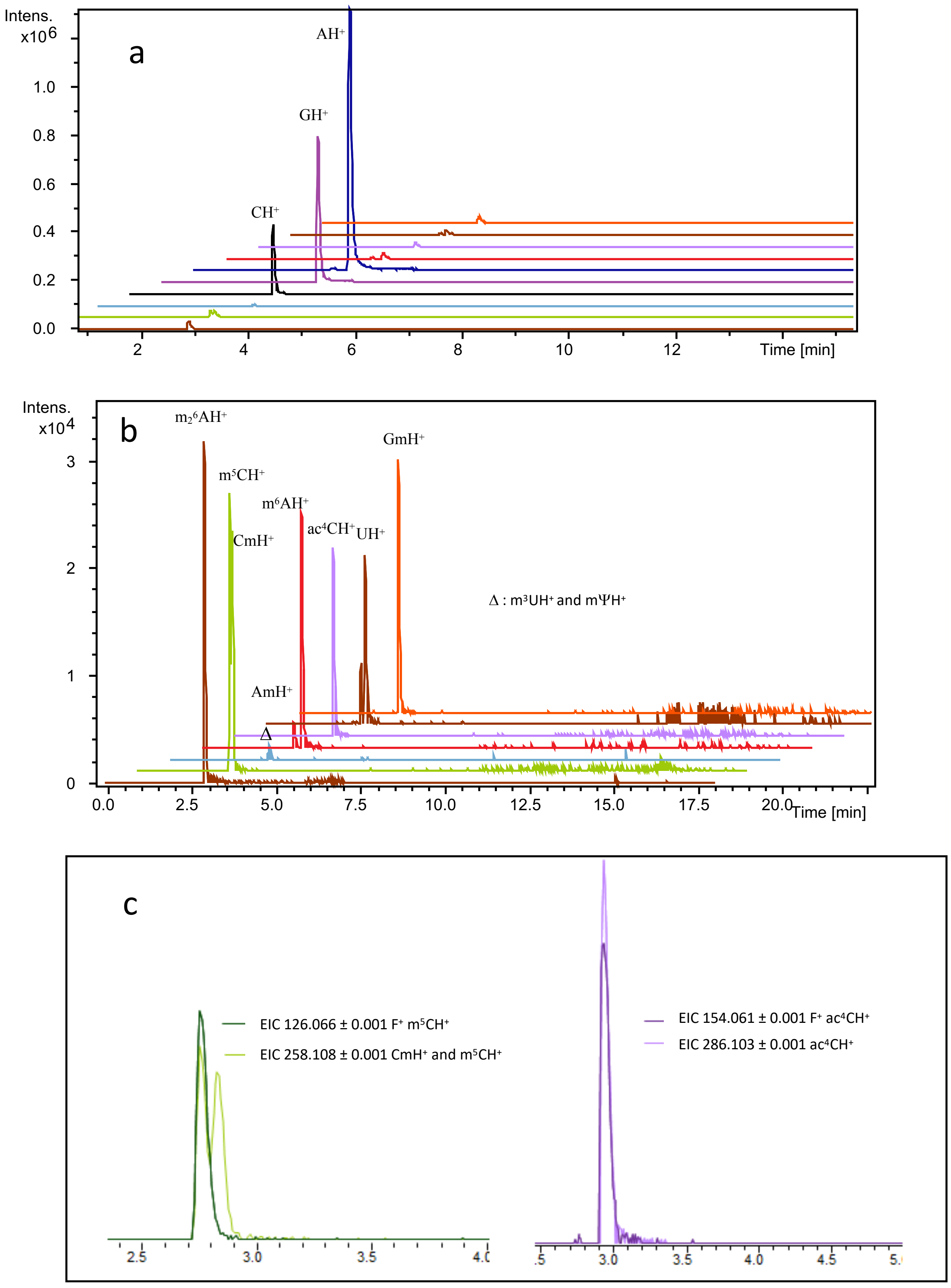

Supplementary Fig. 10: LC-HRMS analysis of nucleosides in Pab-16S rRNA. EIC chromatograms show molecular ions (MH^+). **a**, all the observed nucleosides (Supplementary Table 3). **b**, closeup showing nucleosides observed at lower concentration. **c**, characterization of isomeric nucleosides (m^5CH^+ and CmH^+) (left), and of Ac^4CH^+(right) with both molecular (MH^+) and fragment F^+ (BH_2^+) ions.

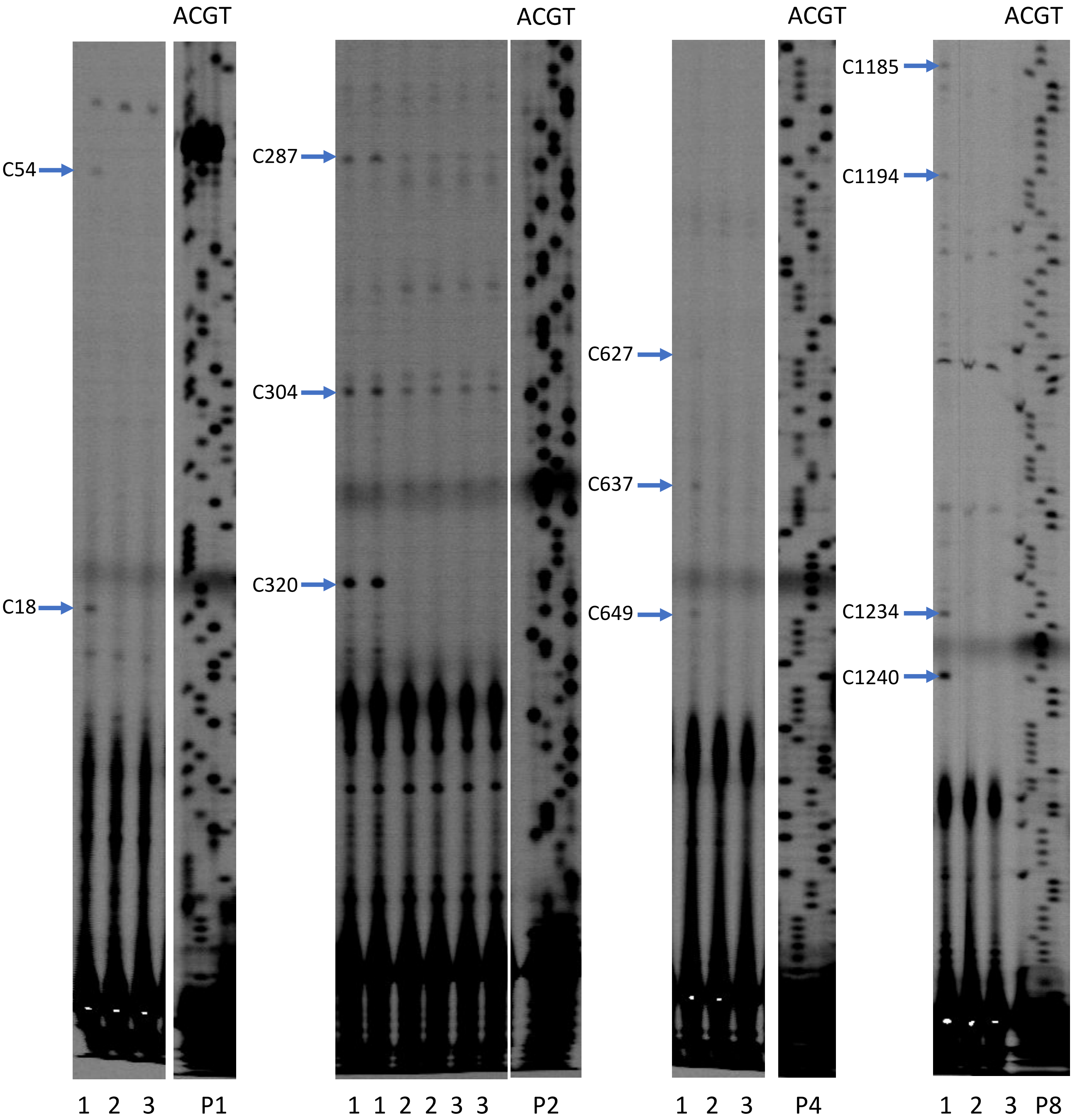

Supplementary Fig. 11: Examples of primer extension analysis of 16S rRNA to map ac^{4}C residues in 16S rRNA. Lane 1, 16S rRNA treated with $NaBH_4$ (100mM, 37°C, 1h), lane 2 control 16S rRNA (37°C, 1h), lane 3, intact 16S rRNA. The RT stop are indicated to the left of the view. The RT primer used is indicated according to the numbering in Supplementary Table 3 (see methods and[4]).

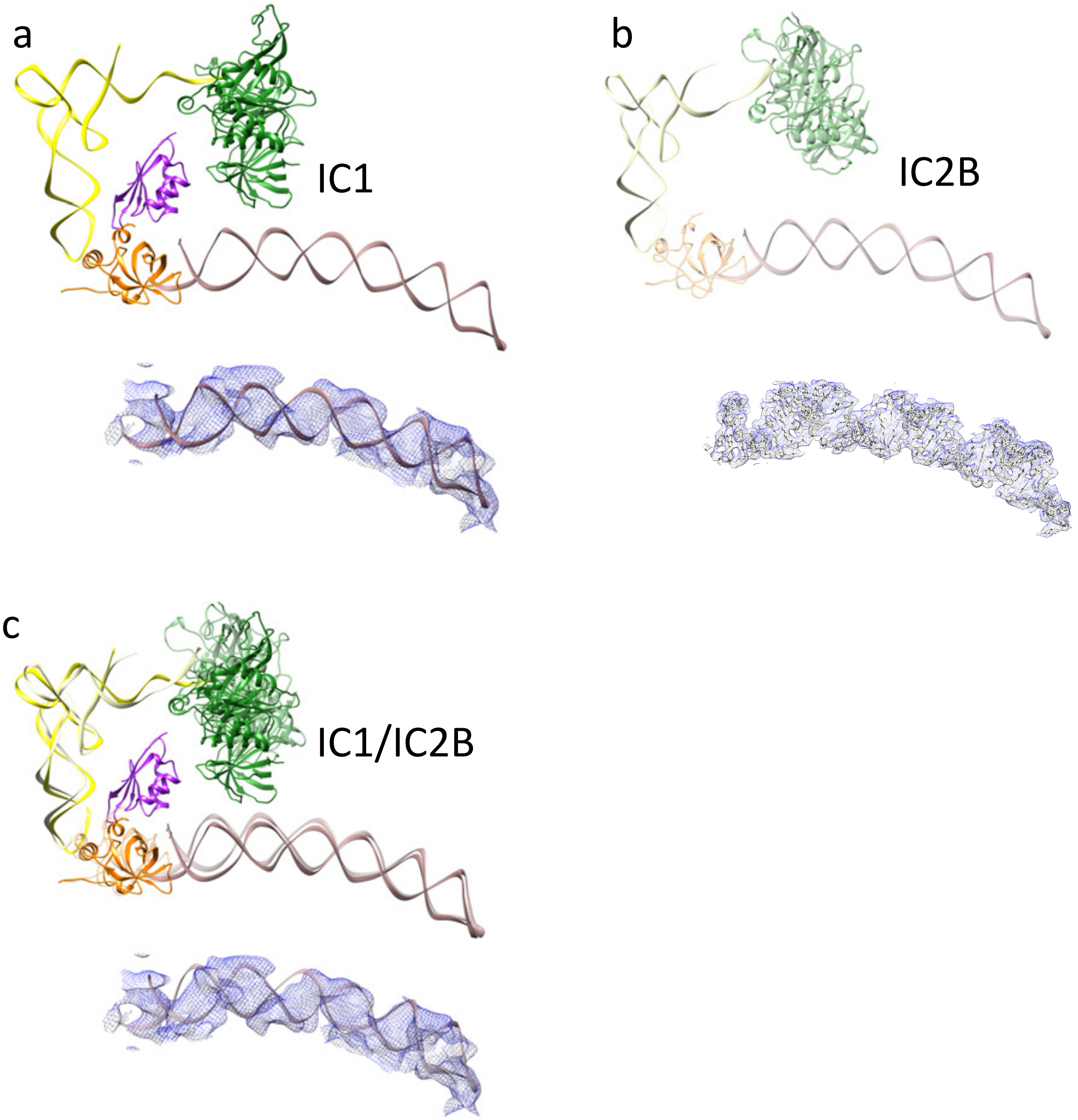

Supplementary Fig. 12: motion of h44 during translation initiation. a, The TC, aIF1, aIF1A and h44 from IC1[5] are shown as cartoon. Electron density for IC1-h44 is represented below. **b**, same for IC2B. **c**, bodies of IC1 and IC2B were superimposed. The view shows the h44 motion associated with the absence of aIF1 in IC2B. The lower part of panel c shows the electron density of IC1-h44 with the IC2B-h44 model. It shows that IC2B-h44 does not fit in IC1-h44 electron density.

Supplementary Tables

		Maps		
		Not masked	Masked	
**Model, map and metrics**	**Reported d_{FSC}**	**Original**	**Original**	**Sharpened**
PDB: 6SW9 IC2A, EMDB-10320				
d_{99}		5.45	5.95	4.3
$d_{model\ (B,Å^2)}$	4.2	3.1 (370)	3 (345)	4.2 (110)
$d_{model\ (B=0)}$		10	10	4.3
d_{FSC_model}		4.9	4.2	4.2
PDB: 6SWC IC2B, EMDB-10322				
d_{99}		4.27	4.36	3.4
$d_{model\ (B,Å^2)}$	3.3	3.3 (150)	3.3 (145)	3.3 (25)
$d_{model\ (B=0)}$		6.9	6.9	3.4
d_{FSC_model}		3.45	3.3	3.3
PDB: 6SWD IC2 body, EMDB-10323				
d_{99}		3.8	3.9	3.2
$d_{model\ (B,Å^2)}$	3.2	3.3 (95)	3.3 (90)	3.2 (10)
$d_{model\ (B=0)}$		4.4	4.4	3.2
d_{FSC_model}		3.3	3.15	3.2
PDB: 6SWE IC2 head, EMDB-10324				
d_{99}		3.7	3.8	3.2
$d_{model\ (B,Å^2)}$	3.1	3.3 (95)	3.3 (90)	3.1 (10)
$d_{model\ (B=0)}$		4.3	4.3	3.2
d_{FSC_model}		3.3	3.1	3.1

Supplementary Table 1: Resolution metrics for datasets IC2A, IC2B, IC2 body and IC2 head. Values of d_{99}, d_{model} (with overall B or B=0) and d_{FSC_model} was calculated for each pair of model/map to assess the quality of the calculated maps as well as the model built in those maps[6].

**Compounds**	**Tr (min)**	**Elemental composition (MH^+)**	**MH^+ (m/z)**	**BH_2^+ (m/z)**
C	2.7	$C_9H_{14}N_3O_5$	244.0928	112.050
Am	2.7	$C_{11}H_{16}N_5O_4$	282.1196	136.061
m^5C	2.8	$C_{10}H_{16}N_3O_5$	258.1084	126.066
Cm	2.8	$C_{10}H_{16}N_3O_5$	258.1084	112.050
A	2.9	$C_{10}H_{14}N_5O_4$	268.1040	136.061
m_2^6A	2.9	$C_{12}H_{18}N_5O_4$	296.1353	164.093*
m^6A	2.9	$C_{11}H_{16}N_5O_4$	282.1196	150.077*
ac^4C	2.9	$C_{11}H_{16}N_3O_6$	286.1033	154.061
G	2.9	$C_{10}H_{14}N_5O_5$	284.0989	152.056
Gm	2.9	$C_{11}H_{16}N_5O_5$	298.1146	164.057*
m^3U	2.9	$C_{10}H_{15}N_2O_6$	259.0924*	127.050
m^1□	2.9	$C_{10}H_{15}N_2O_6$	259.0924*	223.071*$^{\#}$
U	2.9	$C_9H_{13}N_2O_6$	245.0768	113.035
ac^4Cm	2.9	$C_{12}H_{18}N_3O_6$	300.1190*	nd

Supplementary Table 2: Detection of nucleosides in *P. abyssi* 16S rRNA. For each detected nucleoside, the retention time is indicated (Methods). The measured molecular mass is indicated for the MH^+ nucleoside and for the BH_2^+ ion (nucleoside fragment ion derived from the base moiety). *Low intensity; nd : not detected. Gm was detected but not unambiguously observed in IC2B electron density. $^{\#}$:according to the ion fragmentation scheme previously described for pseudouridine[7].

PDB:	State	Ribosome complex	Organism	Sequence mRNA	Position SD-duplex
5JB3	IC0	30S-aIF1-aIF1A-aIF2-tRNA-mRNA	*P. abyssi*	UGGAGGUGAUUUAAAUGCCA	down
5JBH	IC1	30S-aIF1-aIF1A-aIF2-tRNA-mRNA	*P. abyssi*	UGGAGGUGAUUUAAAUGCCA	down
	IC2B	30S-aIF1A-aIF2-tRNA-mRNA	*P. abyssi*	UGGAGGUGAUUUAAAUGCCA	down
5LMN	State-1A	30S-IF1-IF3-mRNA	*T. thermophilus*	GCUCUUUUAACAAUUUAUCAGGCAAGGAGGUAAAAAUGUUCA	up
5LMO	State-1B	30S-IF1-IF3-mRNA	*T. thermophilus*	AGGCAAGGAGGUA	up
5LMP	State-1C	30S-IF1-IF3-mRNA	*T. thermophilus*	GCUCUUUUAACAAUUUAUCAGGCAAGGAGGUAAAAAUGU	up
5LMQ	State-2A	30S-IF1-IF3-mRNA-tRNA	*T. thermophilus*	GCUCUUUUAACAAUUUAUCAGGCAAGGAGGUAAAAAUGUUCA	up
5LMR	State-2B	30S-IF1-IF3-mRNA-tRNA	*T. thermophilus*	GCUCUUUUAACAAUUUAUCAGGCAAGGAGGUAAAAAUGUUCA	middle
5LMS	State-2B	30S-IF1-IF3-mRNA-tRNA	*T. thermophilus*	GCUCUUUUAACAAUUUAUCAGGCAAGGAGGUAAAAAUGUUCA	up
5LMT	State-3	30S-IF1-IF3-mRNA-tRNA	*T. thermophilus*	GCUCUUUUAACAAUUUAUCAGGCAAGGAGGUAAAAAUGUUCA	up
5LMU	State-4	30S-IF3-mRNA-tRNA	*T. thermophilus*	GCUCUUUUAACAAUUUAUCAGGCAAGGAGGUAAAAAUGUUCA	up
5LMV	State-III	30S-IF1-IF2-IF3-mRNA-tRNA	*T. thermophilus*	GCUCUUUUAACAAUUUAUCAGGCAAGGAGGUAAAAAUGUUCA	up
4V51		70S-mRNA-3tRNA-paromomycin	*T. thermophilus*	GGCAAGGAGGUAAAAAUGUUCAAA	no SDaSD
4V4Y		70S-mRNA-P-EsitetRNA	*T. thermophilus*	UUUUUUUUUUUUUUUUUUGGCAAGGAGGUUUUUUUUUUUUUUUUUUUUUUUU	down
4V4Z		70S-mRNA-P-Esite tRNAs	*T. thermophilus*	GGCAAGGAGGUAAAAAUGAAAAAAAAA	middle
4V6G		70S-mRNA-P-Esite tRNAs	*T. thermophilus*	GGCAAGGAGGUAAAAAUGAAAAAAA	up
4WQ1		70S-mRNA-tRNA	*T. thermophilus*	GGCAAGGAGAAAAAAAUGCACAAAAAAAAA	no SDaSD
1JGO		70S-mRNA-3tRNA	*T. thermophilus*	GGCAAGGAGGUAAAAAUUUUUUAAAAAA	down
1JGP		70S-mRNA-3tRNA	*T. thermophilus*	GGCAAGGAGGUAAAAAUUUUUUAAACGUAAAUC	down
1JGQ		70S-mRNA-3tRNA	*T. thermophilus*	GGCAAGGAGGUAAAAAUUUUUUAAACGUAAAUCUACU	down
2E5L		30S-mRNA	*T. thermophilus*	GAAAGAGAAAGA	down

Supplementary Table 3: mRNAs used in various structural studies of bacterial and archaeal complexes cited in the text. The position of the SD duplex in the chamber is indicated (see text). Parts of the mRNA colored in cyan were not modeled. The GGAGG sequence is highlighted in green and the AUG codon is in red.

16S rRNA RT primers	Mapped N^4-acetylcytidines
1-GAGCCGTCCGCCGGTGGCGTC	ac^4C53, ac^4C17
2-GTTTCGCGCCTGCTGCGCCCC	ac^4C319, ac^4C303, ac^4C286, ac^4Cm250
3-TTGCCCAGCCCTTATTCCCGG	ac^4C394, ac^4C379, ac^4C319, ac^4C286
4-GCCGGGCGCCTTCGCCACTGG	ac^4C590, ac^4C626, ac^4C636, ac^4C648
5-CGAAGCTCGCCCGACACCTAG	ac^4C718, ac^4C751
6-GAGGTTCCCGGCGTTGAATCC	ac^4C868, ac^4C851, ac^4C848*, ac^4C839, ac^4C828
7-GACTGGCAACTGGGGGCGCGG	ac^4C957, ac^4C998, ac^4C1028, ac^4C1041
8-CGAGTTGCAGCCCGCGATCCG	ac^4C1147, ac^4C1184, ac^4C1193, ac^4C1233, ac^4C1239

Supplementary Table 4: list of primers used for reverse transcription and the corresponding ac^4C residues identified. The experiments are described in the Methods section. Examples of gel analyses are shown Fig. 2 and Supplementary Fig. 11.

Supplementary references

1. Scheres, S.H. RELION: implementation of a Bayesian approach to cryo-EM structure determination. *J. Struct. Biol.* **180**, 519-530 (2012).
2. Kucukelbir, A., Sigworth, F.J. & Tagare, H.D. Quantifying the local resolution of cryo-EM density maps. *Nature Methods* **11**, 63 (2013).
3. Cannone, J.J. et al. The Comparative RNA Web (CRW) Site: an online database of comparative sequence and structure information for ribosomal, intron, and other RNAs. *BMC Bioinformatics* **3**, 2 (2002).
4. Thomas, J.M. et al. A Chemical Signature for Cytidine Acetylation in RNA. *J Am Chem Soc* **140**, 12667-12670 (2018).
5. Coureux, P.D. et al. Cryo-EM study of start codon selection during archaeal translation initiation. *Nat Commun* **7**, 13366 (2016).
6. Afonine, P.V. et al. New tools for the analysis and validation of cryo-EM maps and atomic models. *Acta Cryst.* **D74**, 814-840 (2018).
7. Basanta-Sanchez, M., Temple, S., Ansari, S.A., D'Amico, A. & Agris, Paul F. Attomole quantification and global profile of RNA modifications: Epitranscriptome of human neural stem cells. *Nucleic Acids Research* **44**, e26-e26 (2015).
