# Supplementary material for: Cryo-EM study of an archaeal 30S initiation complex gives insights into evolution of translation initiation
Source: Commun Biol. 2020 Feb 6;3:58. doi: 10.1038/s42003-020-0780-0 (PMC7005279; doi:10.1038/s42003-020-0780-0)
Supplement: Supplementary file 2 — Description of Additional Supplementary Items [file 42003_2020_780_MOESM2_ESM.pdf]

Description of an additional supplementary item for:

Cryo-EM study of an archaeal 30S initiation complex gives insights into evolution of translation initiation

Supplementary Data 1: Sequence alignment of archaeal aS21.

The supplementary file aS21.fa contains a sequence alignment in the fasta format of 470 archaeal aS21 as identified through BLAST search against archaeal genomes using the *P. abyssi* aS21 sequence as a query. The alignment was manually adjusted.
